# Low Socioeconomic Status Associated with Poor Cancer Screening Perceptions in Malaysia: Analysis of Determinant of Health among General Population

**DOI:** 10.31557/APJCP.2020.21.11.3137

**Published:** 2020-11

**Authors:** Shamsul Azhar Shah, Mohd Ihsani Mahmood, Norfazilah Ahmad

**Affiliations:** 1 *Department of Community Health, UKM Medical Centre, Kuala Lumpur, Malaysia. *; 2 *Disease Control Division, Ministry of Health Malaysia, Health State of Wilayah Persekutuan Kuala Lumpur, Kuala Lumpur, Malaysia. *

**Keywords:** Socioeconomic status, predictors, cancer screening perceptions, general population

## Abstract

**Background:**

The recent data shows reduced uptake on cancer screening where the Perception towards cancer screening by the public is one of the favorable factor might influence the screening uptake. Therefore, this study aims to determine the predictors of poor cancer screening perceptions among the population in Johor, Malaysia.

**Methods:**

This was a cross sectional study of 1,312 respondents selected using a multistage design. Questionnaires relating to the demographic characteristics, socioeconomic profiles, social and physical environment, knowledge and perception of cancer screening were gathered. Multiple logistic regression models were used to examine the variables and their association with poor perceptions of cancer screening.

**Results:**

Overall, 871(66.4%) respondents had poor perceptions of cancer screenings; 68.4% among males and 64.4% among females. In the multivariable analysis in the category of income, the bottom 40% and lower middle 40%, had not subscribed to health insurance, had poor social support, absence of any family history of cancer or comorbid illnesses, no previous attendance for cancer screening and poor knowledge of cancer, all of which were associated with their poor cancer screening perceptions.

**Conclusion:**

One way of developing cancer screening services to detect cancer in its early stage could include efforts to reach people with less awareness about cancer screening tests, lower socioeconomic status, and inadequate social support. Particular consideration should be taken to locate those who never had health insurance or attended cancer screening tests to provide the appropriate resources.

## Introduction

Cancer mortality statistics have dramatically risen in recent years (Zainal Ariffin and Nor Saleha, 2011). A total of 103,507 new cancer cases were diagnosed in Malaysia between 2007and 2011, 46,794 (45.2%) in males and 56,713 (54.8%) in females (source MNCR 2007-2011). This means 1 in 10 males were at risk of getting cancer, while for females, the risk was 1 in 9. Globocan in 2012 estimated 21 700 deaths occurred in Malaysia where 9.2% of the population are at risk of dying from cancer before the age of 75, and in 5-years time, the number of cases will rise to 384.1 per 100 000 population. The five most frequent cancers by total number of cases for both sexes are breast, colorectal, lung, cervix uteri and nasopharynx (Ferley et al., 2013). 

Studies have shown that there is a lack of awareness among the public in taking tests such as pap smear for detecting cervical cancer (Wong et al., 2009), mammograms for breast cancer (Norsa’adah et al., 2011), colorectal (Yusoff et al., 2002) and prostate cancer screening (Hafiz et al., 2016). In addition, it has been reported that while most people nowadays have some knowledge of cancer and cancer screening, unfortunately, the perception of cancer screening still remains low. Consequently, the number of studies geared towards finding what influence the public health is very limited. This is also because most previously conducted studies have focused on healthcare staff, students, cancer patients and also on particular ethnic groups. By studying the public perceptions on cancer screening and taking note of their attitudes towards it, more effective approaches can be created in increasing public participation in cancer screening.

The purpose of this study is to measure the level of public understanding on cancer screening and to understand the factors contributed to the perceptions of the public. These contributing factors have a significant influence in shaping public perceptions which in return, will affect the overall attitude, both negative and positive ones, towards cancer screening. It has been emphasized in the literature that socio-economic position - either individual or in terms of the neighbourhood - acts as the primary exposure to perceptions of health (Kawachi and Subramaniam 2007). 

## Materials and Methods

This cross-sectional study was done in the southern state of the Malay Peninsula, Johor. Focused on the residents of Johor Bahru (the capital district of Johor) as the sample population . The population was identified from the National Population and Housing Census 2010, as updated by the Department of Statistics, Malaysia with the calculated sample size as 1,470 samples. 

*Sample size*

The sample size calculation is based on the criteria below;

a) Prevalent expectations related to perceptions of health screening tests. = 392

perception and considering; Estimated percentage of each factor involved as a predictor variable for that perception.

i) Margin of difference or margin of error (e) = 0.05

ii) Confidence interval or confidence interval at 95%

b) The design effect of the study in the sampling methodology = 1.5

Additionally, sample sizes will be adjusted to;

c) Number of Age-sex estimates = 2

d) Expected percentage of respondents who did not respond to the study (anticipated non-response) = 0.08

For final sample size calculation; 

Sample size = (a*b*c)/0.08= (392*1.5*2)/0.08 = 1,470 

Therefore, the total actual size required in this study was 1,470 after some adjustments were made.

*Participants and Data Collections*

All non-institutionalized individuals, male and female, aged 18 to 65 years were targeted as respondents in this study. The eligibility criteria included: residence of the selected households in the study area, being physically and mentally able to read and understand a consent form and complete the survey instruments. Those suffering from chronic debilitating diseases and actively undergoing cancer treatment were excluded from this study. 

*Instruments*

For this study, the research instrument was a set of self-administered questionnaires that had been validated prior to conducting the study. The validated questionnaire consisted of four sections: Section A is concerned with demography and health status; Section B covers the Interpersonal Support Evaluation List (ISEL) (Cohen et al., 1985); Section C covers Knowledge about cancer screening (Stubbings et al., 2009) and Section D concerns the Cancer Screening Perceptions Scale (CSPS) (Mahmood et al., 2016). All the topics of the questionnaire were reviewed in advance for their appropriateness with regards to the study’s objectives, mode of data collection and the cultural diversity of the study population. 

*Data Analysis*

The data was analyzed using SPSS version 21. All socio-demographic data were presented as nominal data with frequency and percentage to demonstrate the demographic are from which the respondents came to give an overview of their cancer screening perceptions before it was statistically analyzed. Bivariate analyses were done to examine the relationship between the demographic, socioeconomic status, social and physical environment, as well as knowledge of cancer screening to good or poor cancer screening perceptions (outcome). A multiple logistic regression analysis was then used to identify any independent correlates of poor cancer screening perceptions, forcing the variables of interest into the model. All possible methods of interaction were checked. 

A three stage hierarchical multiple regression was conducted with level of cancer screening perception as the dependent variable. Knowledge on cancer screening test, Socioeconomic level and Social support was entered at stage one of the regression to control for other variables responding. All these variables have been selected to be put at block 1 because of highly significant at bivariate analysis. For block 2, the subscriptions of health insurance and attended for cancer screening were entered at stage two. It is due to statistically significant at bivariate analysis and want to see its change in model fitness when merged together with block 1 variables. For variables namely gender, family history of cancer, races, marital status and religions were entered at stage three. All these stages variables were entered in this direction as it seemed chronologically relevant for a person to become aware something especially on their health. Whereas subscription of health insurance and attended for cancer screening at the block 2, occur when a person has perceived on good behavior after become aware for them to sustain their health and life. 

## Results

*Study population*

In this study, 1173 (89.4 %) of the respondents are working people with 349(26.6%) were semiprofessionals while 286 (21.8%) were categorized as lesser level professionals and proprietors of medium-sized businesses. The mean age of respondents was 34 years old, with respondent as young as 19 years to the 64 years old whom involved in this study.

About 578 (44.1%) of the respondents had a monthly income between MYR 1900 to MYR 4499. The median income level for respondents was MYR 3286.30, which indicates that these respondents were relatively wealthy, since the national average monthly income is only MYR 2,312. This shows that, roughly 33% of respondents obtained a salary above the national mean. Concerning their socioeconomic positions based on the Hollinghead Score, the mean score was 55.80, with the majority of respondents falling into a lower socioeconomic position, accounting for 57.9% compared to 42.1% with a high socioeconomic position with almost 50% of them have health insurance, making them excellent potential prospects for improving both the perception of cancer screening and its uptake. Details can be refer to Table 1.

Table 2 also provides an overview of the level of cancer screening perceptions with mean score was 91.55, knowledge of cancer screening tests the mean score was 14.87, indicate average knowledge on cancer screening among the respondents and social support mean score, 24.70 in this study. It describes the mean and standard deviations for all three scores. The level of perception had wide standard deviations, which indicate a large amount of variation in the group being studied. It also indicates that 64.9% of respondents had low perceptions of cancer screening compared to 35.1% with positive perceptions.

*Predictors for Cancer Screening Perceptions*

In this study, the majority of 66.4% of respondents have negative perceptions on cancer screening. Therefore the predictors for cancer screening perceptions discovered were explored using bi-variable analysis and multivariable analysis.

Bivariate analysis correlates of poor perceptions cancer screening included: median income < RM 2414.96 (p<0.001); low socioeconomic position (p<0.001); no health insurance (p<0.001); poor social support (p<0.001); no family history of cancer (p<0.001); no comorbid illness (p<0.001); no attendance at cancer screening (p<0.001) and poor knowledge of cancer (p<0.001). Details can be seen in table 3 where most demographic characteristics were not significantly associated with poor cancer screening perceptions. 

A multiple logistic regression was conducted. For model 1, the categories of income for the bottom 40% and the lower middle 40% and those with no health insurance cover contributed significantly to the regression model. The bottom 40% in the income category, compared to the top 20%, were 12 times more likely to have poor perceptions of cancer screening (95% CI 6.01 - 26.95, p < 0.001). The lower middle 40% in the income category were eight times more likely to have poor cancer screening perceptions compared to Top 20%. Meanwhile, those who did not have health insurance cover were 65% more likely to have poor perceptions of cancer screening compared to those with (OR 1.65, 95% CI 1.24 – 2.19, p < 0.001). 

In Model 2, social environmental factors were added. The results show that poor social support was four times more likely to promote poor cancer screening perceptions as opposed to good social support (95% CI 2.90 – 5.45, p < 0.001). Those without a family history of cancer compared to those who had were 2 times more likely to have poor perceptions of cancer screening (95% CI 1.39 – 3.09, p < 0.05). Adding these social environment predictors variables increased the fit of the model significantly to Model 1 ( χ2 (9) = 538.292, p < 0.001). 

In Model 3, the physical environment factors and knowledge about cancer were examined to discover whether they had any relationship to cancer screening perceptions. The absence of any comorbid illnesses was three times more likely to influence poor cancer screening perceptions than the presence of a comorbid illness (95% CI 1.61 – 4.22, p < 0.001). In addition, those who had not participated in cancer screening programs were 74% more likely to have poor cancer screening perceptions than those who had (95% CI 1.18 – 2.57, p < 0.01). Also, poor knowledge of cancer compared to good knowledge was 5.44 times more likely to result in poor cancer screening perceptions (95% CI 3.84 – 7.72, p < 0.001). All variables are independent of the other predictors. Adding these predictor variables related to poor cancer screening perceptions improved the accuracy of the predictions significantly compared to Model 2 ( *χ*^2^ (12) = 662.898, p < 0.001).

Concerning the predictors of respondents’ poor cancer screening perceptions, significant predictors were found to be associated with the income category bottom 40% and lower middle income 40% categories and respondents who did not have health insurance cover, had poor social support, no family history of cancer, no comorbid illness, had not attended cancer screening and had poor knowledge of cancers. Details are presented in Table 4. 

**Table 1:** Descriptive Data on Demographic Variables, Social Environment

Variables	n (%) n=1312
Gender	
Male	649 (49.5)
Female	663 (50.5)
Race	
Malay	1164 (88.7%)
Chinese	72 (5.5%)
Indian	49 (3.7%)
Others	27 (2.1%)
Marital status	
Married	894 (68.1%)
Divorced	23 (1.8%)
Widowed	32 (2.4%)
Unmarried	363 (27.7%)
Level of Education	
Secondary School(SPM)	467 (35.6%)
STPM/Diploma	324 (24.7%)
Degree	513 (39.1%)
Masters	6 (0.5%)
PhD/Fellowship	2 (0.1%)
Working	
Yes	1173 (89.4%)
No	139 (10.6%)
Level of Occupation	
Higher Executives, Proprietors of Large Businesses, and Major Professionals	41 (3.1%)
Administrators, Lesser Professionals, Proprietors of Medium Sized Businesses	286 (21.8%)
Smaller Business Owners, Farm Owners, Managers, Minor Professionals	209 (15.9%)
Technicians, Semiprofessionals, Small Business Owners	349 (26.6%)
Clerical and Sales Workers, Small Farm and Business Owners	244 (18.6%)
Smaller Business Owners, Skilled Manual Workers, Craftsmen, and Tenant Farmers	6 (0.5%)
Machine Operators and Semiskilled Workers	38 (2.9%)
Farm Laborers/ Service Workers	51 (3.9%)
Not working	88 (6.7%)
Level of Income category (MYR)	
Bottom 20% (<1900)	305 (23.2%)
Lower Middle (1900 – 4499)	578 (44.1%)
Upper Middle (4500 – 9999)	412 (31.4%)
Top 20% (>10000)	17 (1.3%)
Social Position Category	
Low	760 (57.9)
High	552 (42.1)
Health Insurance Cover	
Yes	650 (49.5%)
No	662 (50.5%)

**Table 2:** Descriptive Finding on Social Environment, Physical Environment and Knowledge on Cancer

Variables		n (%) n=1312
Religion	Muslim	1170 (89.2%)
	Buddhist	69 (5.3%)
	Hindu	42 (3.2%)
	Christian	31 (2.4%)
Level of Social Support		
Social Support Category	Low	582 (44.4)
	High	730 (55.6)
Family History Cancer	Yes	190 (14.5%)
	No	1122 (85.5%)
Healthcare Facilities	Yes	1049 (80.0)
	No	263 (20.0)
Comorbid Illness	Yes	183 (13.9)
	No	1129 (86.1)
Comorbid Illness		
Hypertension	Yes	111 (8.5%)
	No	1201 (91.5%)
Diabetes	Yes	82 (6.3%)
	No	1230 (93.8%)
Heart Disease	Yes	7 (0.5%)
	No	1305 (99.5%)
Attended Cancer Screening	Yes	334 (25.5%)
	No	978 (74.5%)
Cancer Screening		
Pap smear	Yes	238 (35.9%)
	No	425 (64.1%)
Mammogram		
Prostatic Specific antigen	Yes	82 (12.4%)
	No	581 (87.6%)
FOBT		
	Yes	6 (0.9%)
	No	643 (99.1%)
	Yes	14 (1.1%)
	No	1298 (98.9%)
Level of Perceptions		
Perceptions Category	Low	871 (66.4)
	High	441 (33.6)
Level of Knowledge		
Knowledge Categories	Good	344 (26.2)
	Poor	968 (73.8)

**Table 3:** Bivariate Analysis for the Related Variable Concerning Cancer Screening Perceptions

Variables	Perceptions Screening		P -value
	Poor (%)	Good (%)	
Age in years *	849 (33.96)	461 (34.22)	0.644
Gender			
Male	444 (68.4)	205 (31.6)	0.124
Female	427 (64.4)	236 (35.6)	
Race			
Malay	774 (66.5)	390 (33.5)	0.171
Chinese	41 (56.9)	31 (43.1)	
Indian	35 (71.4)	14 (28.6)	
Others	21 (77.8)	6 (22.2)	
Marital status			
Married	604 (67.6)	290 (32.4)	0.176
Divorced	15 (65.2)	8 (34.8)	
Widowed	25 (78.1)	7 (21.9)	
Unmarried	227 (62.5)	136 (37.5)	
Income	851 (2414.96)	461 (4894.77)	<0.001
Level of Income category (RM)			
Bottom (<1900)	269 (89.1)	33 (10.9)	<0.001
Lower Middle	448 (83.1)	91 (16.9)	
Upper Middle	125 (28.9)	307 (71.1)	
Top (>10000)	9 (23.1)	30 (76.9)	
Social Position			
Low	605 (79.6)	155 (20.4)	<0.001
High	246 (44.6)	306 (55.4)	
Health Insurance			
No	507 (76.6)	155 (23.4)	<0.001
Yes	364 (56.0)	286 (44.0)	
Religiosity			
Muslim	777 (66.4)	393 (33.6)	0.634
Buddhist	42 (60.9)	27 (39.1)	
Hindu	30 (71.4)	12 (28.6)	
Christian	22 (71.0)	9 (29.0)	
Social Support			
Poor	482 (82.8)	100 (17.2)	<0.001
Good	369 (50.5)	361 (49.5)	
Family History Cancer			
No	769 (68.8)	349 (31.2)	<0.001
Yes	102 (52.6)	92 (47.4)	
Healthcare Facilities			
No	183 (69.6)	80 (30.4)	0.22
Yes	688 (65.6)	361 (34.4)	
Comorbid Illness			
No	755 (66.9)	374 (33.1)	<0.001
Yes	96 (52.5)	87 (47.5)	
Attendance at Cancer Screening			
Never	693 (70.9)	285 (29.1)	<0.001
Ever	178 (53.3)	156 (46.7)	
Knowledge on Cancer			
Poor	753 (77.8)	215 (22.2)	<0.001
Good	118 (34.3)	226 (65.7)	

*, mean(SD); **, Mann Whitney test (median) #P-value refer to chi square and t-test

**Table 4:** Multiple Logistic Regression for Variables Predicting Poor Perceptions of Cancer Screening

Variables	Model 1				Model 2				Model 3			
	B	SE (B)	Adj OR	95% CI	B	SE (B)	Adj OR	95% CI	B	SE (B)	Adj OR	95% CI
Constant	-0.695	0.338	0.499		-1.892	0.669	0.151		-3.756	0.775	0.023	
Income												
Top 20%			1				1				1	
Bottom 40%	2.544	0.383	12.73***	6.01 - 26.95	2.469	0.416	11.81***	5.23 - 26.66	1.919	0.746	6.82**	1.58 - 29.43
Middle B 40	2.085	0.352	8.05***	4.04 - 16.04	2.082	0.383	8.02***	3.79 - 16.99	1.421	0.466	4.14**	1.66 - 10.33
Middle U 40	0.292	0.348	0.75	0.38 - 1.48	0.303	0.38	0.74	0.35 - 1.55	0.459	0.439	0.63	0.27 - 1.49
Health Insurance												
Yes			1				1				1	
No	0.501	0.144	1.650***	1.24 - 2.19	1.38	6.16	1.83***	1.36 - 2.48	0.719	0.169	2.05***	1.47 - 2.86
Social Support												
Good							1				1	
Poor					1.38	0.16	3.98***	2.90 - 5.45	1.179	0.171	3.25***	2.32 - 4.55
Family cancer												
Yes							1				1	
No					0.73	0.203	2.07***	1.39 - 3.09	0.526	0.236	1.69*	1.07 - 2.69
Comorbid Illness												
Yes											1	
No									0.959	0.245	2.61***	1.61 - 4.22
Attendance for Cancer Screening												
Yes											1	
No									0.552	0.197	1.74**	1.18 - 2.57
Knowledge												
Good											1	
Poor									1.694	0.178	5.44***	3.84 - 7.72
Model Coefficient	χ^2^ = 424.361 (4) ***				χ^2^ = 538.292 (9)***				χ^2^ = 662.898 (12) ***			
-2 Log likelihood	1248.979				1135.318				937.628			
Nagelkerk R square	0.384				0.486				0.593			
Hosmer Lemeshow Test	P=0.844				P=0.128				P=0.084			
Classification overall percentage	79.7				81.2				81.8			

Adj OR, Adjusted Odds Ratio; CI, Confidence Interval; χ^2^ ( ) = chi square (df); ***p<0.001; ** p<0.01; *p<0.05; A Multiple Logistic Regression with Backward Logistic Regression was applied; Multicollinearity was checked and none found. No any significant interaction variables were found.

## Discussion

This study measured the potential predictors that could influence cancer screening perceptions. The large and specific sample population studied distinguishes this study from others. The result of this study is highly applicable to the general population, and amendments could subsequently be made to healthcare systems and policies in order to reduce cancer mortality rates and morbidity.

The present study showed that poor cancer screening perceptions among the population were determined by lower levels of income, lack of health insurance, inadequate social support for life changing stress, a family history of cancer, the presence of comorbid illness, prior attendance at cancer screening and knowledge about cancer. 

This study’s results highlight the role of the participants’ socioeconomic level in their cancer screening perceptions. The influence of lower categories of income remained highly significant in the analysis, even after adjustment for the respondents’ variables. Regarding cancer screening perceptions, previous studies have found that people with lower income levels reveal lower perceptions of the value of cancer screening (Damiani et al., 2012; Peretti-Watel et al., 2016). This study generally fits within this pattern, as socioeconomic vulnerability correlated with lower perceptions of cancer screening (Olofsson and Rashid 2011). 

Such understandings of cancer screening when mixed with socioeconomic disparity are common among vulnerable groups who frequently miss health promotion and screening programmes. This is frequently found in residential areas that are distant from urbanized areas, and which have inadequate healthcare facilities (Patel et al., 2014). Poor levels of knowledge are always associated with low socioeconomic groups and this clearly indicates a tendency to low take-up of screening tests for early detection (Damiani et al., 2012). This might be due to low perceptions of cancer screening tests or these tests being perceived as less important than other health issues. 

The current study shows health insurance to be a significant predictor of cancer screening perception. This finding is similar to other studies where lack of health insurance cover was a factor associated with poor take-up of Pap smear screening tests (Farooqui et al., 2013). Lack of prevention and early detection of diseases are also the reasons why health insurance is also lacking, resulting in poor perceptions of the value of screening tests, as expected.

Poor social support is significantly associated with lower cancer screening perceptions, according to this study. The findings are similar to previous findings by Documet et al., 2015, that good social support was associated with mammogram and Pap test compliance as they were perceived as good tools for the prevention of cancer (Documet et al., 2015). In addition, social support has a moderating effect on the relationship between education and Pap test compliance. This work suggests poor social support can result in people having less knowledge about cancer prevention. This view has been supported by other studies, that women aged under 40 years old have poor perceptions of cancer screening and therefore they have poor compliance with Pap smear tests (Lee et al., 2013).

The reason why social support can influence the perception of cancer screening is still a grey area. It has been suggested that social support could act as a stimulant and may lessen the deleterious effect of stressful events (Cohen and Hoberman 1983). It has the ability to relieve the stress associated with cancer screening and it may be able to encourage positive screening behavior.

Individuals with a family history of cancer have a greater likelihood of undergoing cancer screening (Rubinstein et al., 2011), probably due to worry about the possibility of inherited diseases and perceiving cancer screening as a valid tool for use as prevention. The findings from this study suggest that when a person has no family history of cancer, they have a poor perception of the value of cancer screening tests. Most of the people who reported they had no family history of cancer had poor perceptions of cancer screening, as found in the study (Wardle et al., 2015). Lack of information about cancer itself made them less aware of the existence of screening programmes. 

The present study showed a significant association between the absence of comorbid illness and poor cancer screening perceptions. This is supported by other studies where a strong correlation existed between low colorectal cancer screening rates and the absence of comorbid illness (Lukin et al., 2012; Loo et al., 2013). Screening rates among comorbidities significantly increased because these people had already reached the stage readiness for enrollment in screening tests (Lukin et al., 2012). 

Participants with comorbidities were thus likely to take up trial screening testing compared to participants without comorbidities (Ford et al., 2008). The presence of a comorbid illness already made them have regular visits to a clinic or hospital for follow up care. Information about diseases, in particular, cancer, was thus always easily obtainable. Their perceptions of cancer screening were always better than those who rarely attended a healthcare centre.

However, in the current study, those had not attended cancer screening programmes previously were significantly associated with having poor perceptions of cancer screening. Similar findings found that not having Pap smear screening could make the participants susceptible to cervical cancer (Binka et al., 2016). Therefore, perceptions of cancer screening were high among those who had already taken part in Pap smear screening. 

Farooqui et al., (2013) reported that cancer screening tests are important as tools for early cancer detection because treatment can be initiated early. Most respondents agreed that cancer screening tests give more benefits than problems and can help patients decide upon their treatment of choice. Cancer screening can give them a better quality of life and reduce the burden on their carers. More positive views were given by the participants who had experienced cancer screening because they were able to feel and appreciate the less invasive procedures and able to get their results within a short period of time (Binka et al., 2016). 

The findings from this study show that the level of knowledge regarding cancer screening makes a significant contribution to cancer screening perceptions. Similar findings were obtained from a study by Bansal et al., 2015, where suboptimal levels of knowledge regarding cancer lead to lower perceptions of cancer screening tests as useful tools (Bansal et al., 2015). Limited knowledge about its advantages also reduced the willingness to participate in testing due to lower perceptions of cancer screening benefits (Kim et al., 2014).

This lack of knowledge is mainly due to inadequate population-based health promotion and screening programmes, poor understanding of cancer risk factors, incorrectly identifying certain conditions as symptoms of cancer, being unaware of existing screening methods and unsure of the available best screening facilities for detecting cancer, and even misconceptions about cancer treatment itself (Dubai et al., 2012; Loo et al., 2013).

To improve the general populace’s perception of cancer screening requires a multifaceted approach and involves several aspects of human belief and behavior which cannot be summarized in a model based on measureable demographic, psychological or social variables. Regardless of the variety of population variables contemplated in this study, the multilevel logistic regression model could not predict > 60% of cancer screening perception outcomes. Furthermore, cancer screening behavior will always be influenced by a range of subjective factors such as coping ability, social expectations, personal beliefs and perceptions. However, the identification of the predictors of cancer screening perceptions produced by the current study may be useful in developing a model that can more precisely predict cancer screening uptake and so encourage greater uptake. 

Generally, perceptions about cancer screening and the acceptance of its need in terms of early detection remain unsatisfactory. The CSPS scales, which contain several constructs from the health belief model, were used to identify and explain Malaysians’ apparent unwillingness to undergo cancer screening (Mahmood et al., 2016). The concept of the health belief model could be applied to measure cancer screening perceptions through its capacity to explain more about preventative health behaviors (Ben-Natan and Adir, 2009). The constructs have generally been found to predict participation in cancer screening based on perceptions about taking action to prevent, screen or control disease conditions (Champion and Skinner, 2008). 

*Strengths*

There are several positive outcomes from this study. Most importantly, the large sample size in this study gives a better representation of the overall population, reducing the effect of extreme or outlier observations. A reasonably large sample size is necessary to produce results that are significant and the high response rate achieved minimizes the risk of non-response bias and increases the likelihood of to producing useful results. 

In addition, the present research used a strong and comprehensive CSPS scale to measure the outcomes of cancer screening perceptions. Its high reliability and validity indices prove that this tool was appropriate for use in this study due to its suitability for the population of interest recruited in this study. 

The general advantage of the 6 point interval scale used in the questionnaire is that the respondents record the magnitude to which they agree (or disagree) with a series of statements about their perceptions of cancer screening. Another strength of this study was the survey or quantitative measure used for a larger sample to discover how people perceived certain issues and the applicability of the findings by extrapolation to a larger population. Subsequently, the findings can help to improve and support the limitations of generating such data from in depth interview and focus group discussions alone.

There are several limitations found in this study, even though the research achieved its aims. This study was primarily limited by the unequal distribution of demographic respondents. The sample of respondents presented a wide gap in frequency in terms of ethnicity and religion. Ideally, the number of participants should have been more evenly distributed by gender as well, therefore the outcomes may not represent the population overall and may not be generalisable. In addition, a larger sample with more diversity would have improved this study’s findings. 

Secondly, the data collected from respondents as a measure of their perceptions only occurred at one point in time. So, while the perceptions of the respondents may be significant during the period studied, their perceptions could change over the time or have been different on another day. 

There are still gaps in the knowledge base that need to be filled because there no direct connection between health behavior, cancer incidence and the perceptions of cancer screening has been found. While the objectives of this study specifically focused on perceptions, this limitation still remains as a flaw as it cannot be proved whether perception is truly related to health seeking behaviour or vice versa. However, this finding is still significant through the correlation between perception level and its predictors.

In conclusion, lower income categories without health insurance cover, poor social support, no family history of cancer, no comorbid illness, did not attend cancer screening tests, together with poor knowledge about cancer are significant predictors of poor perception of cancer screening programmes in this study. Designing specific health educational cancer programmes with sub-components involving targeted groups within the population and media support are important for promoting screening uptake to change the negative perceptions of cancer screening to positive ones. This, in turn, may provide people with accurate personal risk perceptions and help them overcome barriers to screening. This can subsequently improve cancer screening behavior and cancer screening uptake and reduce the number of deaths due to cancer in the long run. 
